# Time in Nature Associated with Decreased Fatigue in UK Truck Drivers

**DOI:** 10.3390/ijerph18063158

**Published:** 2021-03-18

**Authors:** Daniel P. Longman, Colin N. Shaw, Veronica Varela-Mato, Aron P. Sherry, Katharina Ruettger, Mohsen Sayyah, Amber Guest, Yu-Ling Chen, Nicola J. Paine, James A. King, Stacy A. Clemes

**Affiliations:** 1 School of Sport, Exercise and Health Sciences, Loughborough University, Loughborough LE11 3TU, UK; V.Varela-Mato@lboro.ac.uk (V.V.-M.); A.P.Sherry@lboro.ac.uk (A.P.S.); K.B.Ruettger@lboro.ac.uk (K.R.); M.Sayyah@lboro.ac.uk (M.S.); A.Guest@lboro.ac.uk (A.G.); Y.Chen2@lboro.ac.uk (Y.-L.C.); N.J.Paine@lboro.ac.uk (N.J.P.); J.A.King@lboro.ac.uk (J.A.K.); S.A.Clemes@lboro.ac.uk (S.A.C.); 2 Department of Anthropology, University of Zurich, 8050 Zurich, Switzerland; cshaw111@me.com; 3 National Institute for Health Research (NIHR) Leicester Biomedical Research Centre, University Hospitals of Leicester NHS Trust and University of Leicester, Leicester LE5 4PW, UK

**Keywords:** driving, HGV driving, long-distance driving, truckers, self-reported fatigue, nature, health and wellbeing, Covid-19

## Abstract

Heavy goods vehicle (HGV) driving is recognised as a highly hazardous occupation due to the long periods of sedentary behaviour, low levels of physical activity and unhealthy food options when working. These risk factors combine with shift work and concomitant irregular sleep patterns to increase the prevalence of fatigue. Fatigue is closely linked with stress and, subsequently, poor physiological and psychological health. In parallel, a wealth of evidence has demonstrated the health and wellbeing benefits of spending time in nature. Here, we sought to examine whether spending time in nature was associated with lower levels of fatigue, anxiety and depression in HGV drivers. 89 long-distance drivers (98.9% male, mean ± SD age: 51.0 ± 9 years, body mass index: 29.8 ± 4.7 kg/m^2^) participating in a wider health promotion programme reported time spent in nature (during and before the Covid-19 pandemic) and symptoms of occupational fatigue, depression and anxiety. After controlling for covariates, truck drivers who visited nature at least once a week exhibited 16% less chronic fatigue prior to the pandemic, and 23% less chronic fatigue and 20% less acute fatigue during the pandemic. No significant differences were observed for either anxiety or depression. As fatigue has a range of physical and mental health sequelae, we propose that increased exposure to natural settings may make a valuable contribution to interventions to promote the health and wellbeing of this underserved group.

## 1. Introduction

### 1.1. Health Problems Associated with Long-Distance Driving

Heavy goods vehicle (HGV) driving is recognised as a highly hazardous occupation. Inherent health-related risk factors include prolonged periods of sedentary behaviour (sitting), low levels of habitual physical activity and unhealthy food options when working. Shift work and long and/or inconsistent working hours combine with tight delivery schedules to underpin a growing body of epidemiological evidence suggesting that HGV drivers are at high risk of developing stress and associated diseases [1]. The World Health Organization recognises the threat to health posed by stress, describing it as the ‘health epidemic of the 21st Century’ [2]. The negative effects of stress are far-reaching, with profound consequences for population health and wellbeing. In 2018, the majority of UK adults (74%) were at some point overwhelmed by stress and unable to cope [3]. The impending global mental health crisis, predicted by the United Nations to arise from the Covid-19 pandemic [4], will exacerbate health and wellbeing problems for all, and particularly for groups with unhealthy working conditions such as HGV drivers.

Physiologically, stress leads to disturbances in the two components of the stress-response system: the autonomic nervous system and the hypothalamic-pituitary-adrenocortical (HPA) axis [5]. The autonomic nervous system represents a balance of the sympathetic nervous system (SNS) and the parasympathetic nervous system (PNS). In response to stress, SNS activity increases, mobilising glucose for energy by promoting catabolic tissue breakdown and metabolism [6]. This facilitates arousal and alertness (the *fight-or-flight* response). In contrast, the PNS is active during periods of rest, and acts to stimulate recovery, healing and immune function (the *rest-and-repair* response) [7,8]. This acute stress response to a threat has adaptive value, promoting survival and preservation of core physiological function. However, it becomes detrimental if this response is chronic. Chronic stress is highly damaging to heath and is recognised as a causative factor in many clinical conditions including impaired mental [9,10], cardiovascular [11,12], cardiometabolic [13,14] and musculoskeletal [15,16] health. Unfortunately, HGV drivers are occupationally exposed to a variety of stressors, including tight delivery schedules, road traffic delays, and the unpredictable behaviours of other road users. This leads to the development of chronic stress [17,18], and health suffers as a result.

Fatigue, defined as “...inefficient action patterns; declining interest, involvement and commitment; reduced concentration and motivation; and negative emotions” [19], is closely related to physiological and psychological stress [20,21]. Recent evidence suggests the relationship between the two is reciprocal in nature; the occurrence of one can be predicted by the occurrence of the other, with sleep quality acting to attenuate the effect of each [22]. The challenging working hours within the logistics and transport industry necessitate irregular sleep patterns, leading to driver fatigue and decreased cognitive performance [23,24]. The consequent reduced vigilance, decision making ability and sensorimotor processing speed all impair a driver’s ability to maintain a correct road position, a constant speed or respond appropriately to changing conditions [25,26]. As a result, fatigue is a significant contributor to crashes [27]. Driving for more than eight hours has been found to double the risk of an accident [28], and insufficient sleep is suggested as responsible for the “disproportionately high number of fatigue-related accidents” involving drivers of HGVs in the UK [29].

The deleterious consequences of chronic fatigue extend far beyond reduced cognition and increased likelihood of an accident [30]. Through considerations of a range of mechanisms, including increased SNS activation [31], hormonal imbalances and the resultant increase in caloric intake [31], a large body of research has linked fatigue to poor cardiometabolic [18,32], cardiovascular [11,12,33] and musculoskeletal [15,16] health. Fatigue also increases all-cause mortality and is linked to an increased incidence of cancer [34,35].

Like stress, fatigue has numerous mental health sequelae [36]. Up to two thirds of people reporting fatigue lasting more than 6-months also suffer from a comorbid psychological disorder, with anxiety and depression featuring prominently [37,38]. In fact, so close is the association that chronic fatigue is argued to be a *forme fruste* of anxiety and depression [39]. The downstream effects of anxiety and depression are insidious and far-reaching, extending beyond psychological wellbeing and function to somatic health. The somatic sequelae of anxiety and depression are often over-represented in HGV drivers [40,41,42,43,44], and include increased general mortality, heart disease, hypertension, stroke, diabetes, Alzheimer’s disease, obesity and cancer. These somatic consequences are, in part, due to disrupted autonomic nervous system and HPA axis function [45]. The plethora of negative health outcomes associated with HGV driving result in, perhaps unsurprisingly, a significantly lower life expectancy than the population average [46].

### 1.2. Health Benefits of Exposure to Nature

Nature, in contrast to HGV driving, is recognised for its ability to provide positive health outcomes [47,48,49]. Spending time in nature influences both components of the stress response system by upregulating PNS activity while downregulating SNS activity, and reducing activity within the HPA axis [50,51]. This induces a physiological state of relaxation and recovery from previous stress [52,53]. As a result, nature provides myriad benefits to both physiological and psychological health and wellbeing. To date, evidence has emerged linking nature exposure with reduced physiological stress, (as measured by sympathetic nervous system activation, e.g., decreased blood pressure), improved heart function, immune function, eyesight, pain control, post-operative recovery and general health, as well as reduced obesity, cardiovascular disease, diabetes, musculoskeletal disorders, asthma/allergies and reduced general mortality [47,48,49]. From a psychological perspective, nature is linked to reduced symptoms and clinical outcomes of both psychological stress and mental health, including anxiety and depression [47,48,49].

Nature exposure therefore represents a promising remedy for many of the negative health outcomes associated with HGV driving. In addition to the abovementioned benefits, nature reduces mental fatigue [54,55,56]. A potential explanation is that nature contains elements which encourage effortless cognitive function, thereby rejuvenating the brain and increasing one’s ability to direct attention towards a particular task [54,55,57]. This rejuvenating effect, and the associated reduction in mental fatigue, has been demonstrated under experimental conditions following short bouts of moderate walking in natural settings [58].

Despite the well-documented hazards of HGV driving, drivers remain underserved in terms of health promotion efforts [59]. As a growing body of literature suggests that spending time in nature benefits health and wellbeing, we sought to test whether an association existed between spending time in nature and levels of acute fatigue, chronic fatigue, and symptoms of anxiety and depression both during and before the Covid-19 pandemic in a sample of HGV drivers.

## 2. Materials and Methods

### 2.1. Study Design, Setting and Participants

Participants for this study were part of a larger cohort of long-distance HGV drivers taking part in a programme of research designed to evaluate the effectiveness of a ‘Structured Health Intervention For Truckers (SHIFT)’. The full protocol of this cluster randomised controlled trial (RCT) has been reported elsewhere [60]. In brief, 386 participants (98.7% male, mean (± SD) age: 47.8 ± 9.4 years; BMI 30.4 ± 5.1 kg/m^2^) were recruited from 25 depots (from one large logistics and transport company) from the Midlands region of the United Kingdom (UK). Posters and information sheets advertising the study were distributed to drivers within participating depots; this was followed by specific recruitment visits by the research team to each site, where drivers could ask any questions regarding the study. During these visits, drivers interested in taking part completed an opt-in form [60]. To be included in the trial, drivers were required to be free from cardiovascular diseases, haemophilia, blood-borne viruses, and have no mobility limitations that prevented them from increasing their daily physical activity levels (physical activity was the primary outcome in the SHIFT trial). The main trial incorporated an internal pilot involving participants recruited from six depots. The measurements taken from participants at baseline as part of the RCT which are relevant to the analyses reported herein are described below (see Baseline measurements), for the participants included in this paper, their baseline measurements took place between February and July 2019.

During the first wave of the Covid-19 pandemic, and the first national lockdown in the UK (March–June 2020), an online survey was created and distributed to participants within the SHIFT Study to examine the impact of the pandemic on this occupational group. At the time of the survey, 220 participants remained in the SHIFT trial and were contacted via the study’s text messaging service and invited to participate in the survey (data collection had been completed in the six pilot sites, and one site had withdrawn from the study). Ethical approval was obtained from the Loughborough University Ethics Approvals (Human Participants) Sub-Committee (reference: R17-P063) for the SHIFT RCT, and further approval was obtained from this committee for the inclusion of this additional online survey (reference: 2020-1444-1221). Written informed consent was obtained from all participants before baseline measurements were taken in the main trial and participants provided informed consent online ahead of beginning the survey.

### 2.2. Online Questionnaire

Participants were invited to complete an online survey, distributed via the ‘Online Surveys’ platform, between May and July 2020. As part of the survey, participants self-reported their weight (kg), number of years worked as a HGV driver, current working hours or furlough status, and sleep duration (hours/day) in the past 14 days. Current body mass index (BMI) was estimated using participants’ height measured at baseline (see measurements section below) and their self-reported weight in the online survey. Symptoms of anxiety and depression during the pandemic were assessed using the validated Hospital Anxiety and Depression Scale (HADS) questionnaire [61]. This questionnaire consists of two subscales, each containing seven questions which assess symptoms of anxiety and depression. Answers to each question are scored on a scale ranging from 0 to 3, with possible total scores for each construct ranging from 0 to 21. For each construct, a score of 7 or less is classified as no symptoms while scores of 8–10, 11–14 and 15–21 are classified as mild, moderate and severe symptoms of anxiety and/or depression, respectively [62]. Work-related chronic and acute fatigue during the pandemic were assessed using the validated Occupational Fatigue Exhaustion/Recovery Scale (OFER-15) [63,64]. This questionnaire contains five questions relating to chronic fatigue and five questions relating to acute fatigue. Responses to each question are scored on a scale ranging from 0 to 6, total scores for each scale are processed and presented on a scale ranging from 0 to 100, with the higher the value indicating a greater degree of acute and/or chronic fatigue.

Questions relating to time spent in nature were also included in the online questionnaire. Specifically, participants reported whether they habitually spent time in nature before the pandemic, they were also asked whether they were spending time in nature during the pandemic. Nature was defined as spaces such as gardens, parks, sports fields, allotments, woodland, lakes, rivers, coastline, beaches or mountains. Participants also indicated the frequency in which they spent time spent in nature, before and during the pandemic, using the following options: no time in nature; once/week; 2–3 times/week; almost every day; every day.

### 2.3. Baseline Measurements

Full details of all measurements taken as part of the SHIFT trial are described elsewhere, however those relevant to the present analyses are outlined here [60]. Baseline data collection took place within the worksites of participating depots and involved a two-hour health assessment occurring at the beginning of participants working shift. All measurements were undertaken by trained research staff and a standardised protocol was followed. Anthropometric measurements included height (measured using a portable stadiometer) and body mass (measured using Tanita DC-360S body composition scales). BMI was subsequently calculated as kg/m^2^.

As part of the health assessment, participants completed a comprehensive questionnaire enquiring about demographic and work-related information (for example, age, sex, education, marital status, years working as a HGV driver, hours spent working per week), lifestyle behaviours (for example, diet, smoking and alcohol intake) and medication use. Symptoms of anxiety and depression were assessed using the HADS questionnaire [61] and work-related chronic and acute fatigue were assessed using the OFER-15 scale [64].

Participants were issued with two accelerometers to wear over an 8-day period following the health assessment. Whilst wearing the accelerometers, participants were also requested to complete a daily log where they recorded sleep onset time (explained to the participant as ‘lights out’), wake-up time, out of bed time, working periods and any non-wear periods of either device.

Physical activity was assessed using the activPAL3 micro accelerometer (PAL Technologies Ltd., Glasgow, UK). This device provides a valid measure of posture and activity in adults [65], and provides a more accurate measure of physical activity in occupational drivers in comparison to waist-worn accelerometers [66]. The activPAL was waterproofed (using a nitrile sleeve and Hypafix [BSN Medical] dressing) and worn on the midline anterior aspect of the upper thigh of participants’ non-dominant side (attached using a second piece of Hypafix dressing). activPALs were initialised and downloaded using manufacturer proprietary software (activPAL Professional v.7.2). Event files were generated and processed using the freely available Processing PAL software (https://github.com/UOL-COLS/ProcessingPAL, version 1.3, University of Leicester, (Leicester, UK)). This software uses a validated algorithm to determine waking wear time [67]. For the purposes of this study, a valid day was defined as >10 h of waking wear, with >1000 steps/day, and <95% of time spent in any one behaviour (e.g., sitting, standing, or stepping). Daily step counts and time in light physical activity (LPA) and moderate-to-vigorous activity (MVPA) were extracted for use in the present analyses, where time in MVPA was derived using a step cadence of >100 steps per minute in bouts of one minute. LPA was calculated by subtracting sitting, standing and MVPA time from waking wear time.

Sleep was assessed using a tri-axial accelerometer (GENEActiv, ActivInsights Ltd., Kimbolton, UK) worn on the non-dominant wrist continuously for 8-days. Accelerometer files were processed in the R package GGIR version 1.8-0 [68] to generate sleep outcome variables, with sleep duration used in the analyses reported in this paper. Sleep periods were informed by the self-reported sleep onset time and out of bed time provided by the daily log data (described above). Where sleep log data were missing, automated ‘sleep windows’ were detected from the accelerometer data using a validated algorithm [69]. Sleep durations identified between self-reported, or algorithm defined, sleep onset time and out of bed time were calculated using a validated sleep detection algorithm [70]. A wear time of ≥16 h over a 24-h period was required to determine a valid night of sleep. Individual nights with a sleep window of >13 h or <2 h, or sleep durations >12 h or <1 h were identified as erroneous and removed.

### 2.4. Statistical Analysis

Independent samples *t*-tests were used at baseline to compare the study subsample to the wider SHIFT sample, to compare nature exposure and outcome variables between drivers allocated to the intervention and control arms, and to compare the outcome variables in participants who visit nature to those who did not. One-way analysis of variance (ANOVA) was performed to determine whether significant differences in outcome variables (chronic fatigue, acute fatigue, anxiety and depression) between individuals grouped by the frequency of their visits to nature were present. Multiple regression was used to determine the potential effect of covariation with other variables (sleep duration, weekly working hours, daily step count, MVPA, LPA, group, i.e., control/intervention) in the relationship between outcome variables and time spent in nature. Finally, hierarchical multiple regression allowed investigation of the ability of time in nature to predict outcome variables, adjusting for covarying factors. All analyses were performed using SPSS v25, with significance set at *p* < 0.05. Initial analyses found that there were no differences in any of the key variables between the intervention and control groups, therefore the sample were analysed as a whole group.

## 3. Results

Of the 220 participants who remained in the trial at the start of the pandemic, 89 (40% response rate from those invited) completed the online survey during the pandemic. The descriptive characteristics of the participants included in the present analyses are reported in Table 1. Baseline characteristics of the 89 participants (42 of whom were in the intervention arm) included in this paper did not differ significantly to the remainder of participants within the SHIFT RCT, suggesting this sub-sample are largely representative of the wider cohort. Specifically, there were no differences at baseline between participants completing the online survey, and the wider cohort in terms of age, duration working as a HGV driver, hours worked per week, BMI, self-reported symptoms of anxiety and depression, acute fatigue, physical activity levels and sleep duration (*p* > 0.05).

### 3.1. Pre-Covid-19 Outbreak (February–July 2019)

Before the Covid-19 outbreak, baseline data revealed that 48.3% (43 of 89) of the participants reported visiting nature 1–3 times a week, 23.6% (21 of 89) visited nature 4+ times per week and the remaining 28.1% (25 of 89) did not visit nature at all. Of the time spent in nature each week by the study cohort as a whole, 41.3% (149.1 h) was in a garden/allotment, 23.2% (83.8 h) in a park/sports field, 13.3% (48 h) in woodland, 7.1% (25.5 h) in hills, 4.8% (17.25 h) on/near water (e.g., pond, lake, river, coastline), and 7.9% (28.5 h) other (e.g., open countryside).

**Figure 1:**
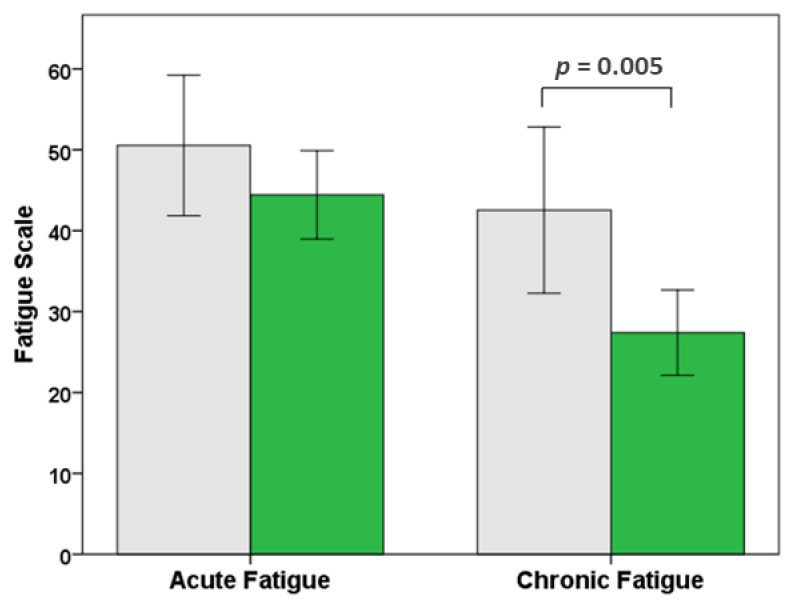
Acute fatigue and chronic fatigue pre-Covid-19. Drivers who visited nature at least once a week (green bars) had significantly lower chronic fatigue than those who did not (grey bars). 95% confidence intervals.

Independent samples *t*-tests were used to investigate the association between spending time in nature and the key outcome measures of chronic fatigue, acute fatigue, anxiety and depression (sexes combined). The results indicated that participants who visited nature at least once a week had significantly lower levels of chronic fatigue than those who did not (Figure 1). There were no significant differences for acute fatigue, anxiety or depression (Table 2).

**Figure 2:**
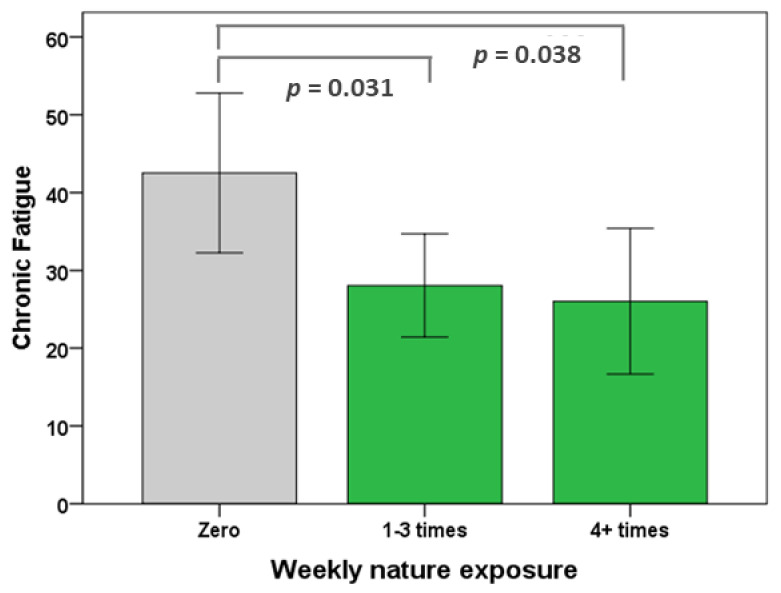
Chronic fatigue pre-Covid-19. Drivers who visited nature at least once a week had significantly lower chronic fatigue than those who did not, but increasing the frequency of nature exposure did not reduce chronic fatigue further. 95% confidence intervals.

The association between frequency of nature visits and chronic fatigue (the only outcome variable achieving statistical significance) was investigated via an ANOVA, with participants grouped as no weekly visits (*n* = 25), 1–3 weekly visits (*n* = 43) and 4+ weekly visits (*n* = 21). There was a statistically significant difference between the groups F(2,86) = 4.186, *p* = 0.018. A Tukey post hoc test revealed that chronic fatigue was lower in the 1–3 weekly visit group (28.06 ± 21.59, *p* = 0.031) and the 4+ weekly visits group (26.03 ± 20.62, *p* = 0.038) than for the no visits group (42.53 ± 24.86). There was no significant difference between the 1–3 weekly visits and the 4+ weekly visits groups (Figure 2).

A multiple regression was performed to consider the potential effect of covariation with other variables in the relationship between chronic fatigue and time spent in nature. A number of potential confounding variables were considered, including anxiety, depression, sleep duration, weekly working hours, daily step count, MVPA, LPA, group (control/intervention) and nature exposure. Of these, weekly working hours (*p* = 0.012), anxiety (*p* < 0.001), daily step count (*p* = 0.022), MVPA (*p* = 0.020), LPA (*p* = 0.030) and whether or not an individual visited nature each week (*p* = 0.001) contributed significantly to the prediction.

A hierarchical multiple regression, adjusting for these factors, was then conducted to investigate the ability of time in nature to predict chronic fatigue. In the first step of the hierarchical multiple regression, weekly working hours, anxiety, daily step count, MVPA and LPA were entered as predictors. This model was statistically significant F(5,79) = 6.583; *p* < 0.001, and explained 29.4% of the variance in chronic fatigue (Table 3). After entry of time in nature at Step 2 the total variance explained by the model increased to 38.1% F(6,78) = 7.995; *p* < 0.001. The introduction of time in nature thereby explained an additional 8.7% of the variance in chronic fatigue (R^2^ change = 0.087; F(1,78) = 10.923; *p* = 0.001). Compared with no weekly visits, spending time in nature was associated with a reduction in chronic fatigue of 15.8 percentage points (see Table 3).

### 3.2. During the Covid-19 Outbreak (May–July 2020)

The pre-Covid-19 analyses were repeated for the time period spanning May to July 2020 among drivers who were still working (*n* = 71, i.e., not currently furloughed due to the Covid-19 pandemic).

**Figure 3:**
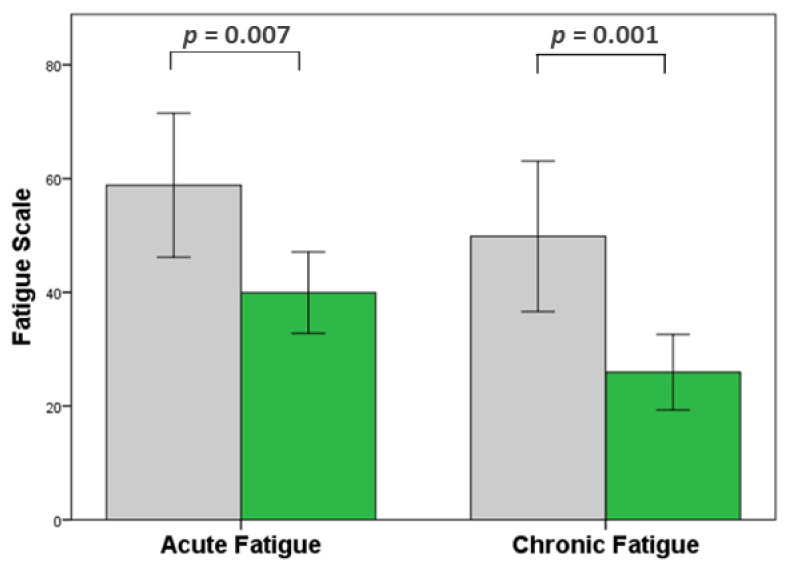
Acute fatigue and chronic fatigue during the Covid-19 outbreak. Drivers who visited nature at least once a week (green bars) had significantly lower acute and chronic fatigue than those who did not (grey bars). 95% confidence intervals.

Independent samples *t*-tests were used to investigate the association between spending time in nature and the key outcome measures. The results indicated that participants who visited nature at least once a week during the pandemic had significantly lower levels of both chronic and acute fatigue than those who did not (Table 4 and Figure 3).

**Figure 4:**
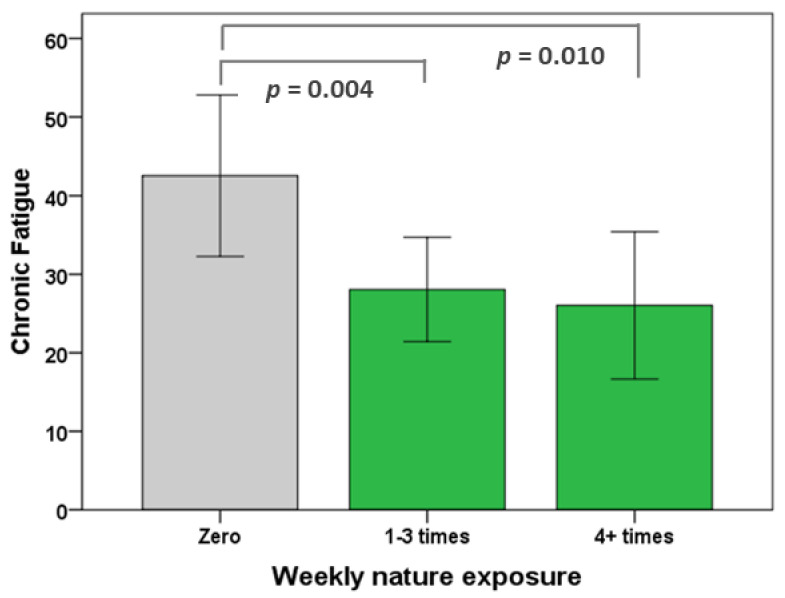
Chronic fatigue during Covid-19. Drivers who visited nature at least once a week had significantly lower chronic fatigue than those who did not, but increasing the frequency of nature exposure did not further reduce chronic fatigue. 95% confidence intervals.

As in the pre-Covid-19 analyses, the effect of frequency of nature visits on chronic fatigue was investigated via an ANOVA. There was a statistically significant difference between the groups (F(2,68) = 6.474, *p* = 0.003), with chronic fatigue being significantly lower in both the 1–3 weekly visit group (*n* = 28, 25.48 ± 21.38, *p* = 0.004) and the 4+ weekly visits group (*n* = 23, 26.52 ± 26.60, *p* = 0.010) than the no visits group (*n* = 20, 49.83 ± 28.29). There was no significant difference between the 1–3 weekly visits and the 4+ weekly visits groups (see Figure 4).

A further multiple regression was performed to consider the relationship between the potential confounding effects of anxiety, depression, sleep duration, weekly working hours, group (control/intervention) and nature exposure on chronic fatigue (due to the restrictions imposed on the study design by Covid-19, we were unable to repeat pre-Covid-19 measures of physical activity). Only anxiety (*p* = 0.019) and whether an individual visited nature each week (*p* < 0.001) contributed significantly to the prediction.

A hierarchical multiple regression was then conducted to investigate the ability of time in nature to predict chronic fatigue, after controlling for anxiety. In the first step of the hierarchical multiple regression, anxiety was entered as a predictor. The model was statistically significant F(1,69) = 33.232; *p* < 0.001, and explained 32.5% of the variance in chronic fatigue (Table 5). After entry of time in nature at Step 2 the total variance explained by the model was 46.7% F(2,68) = 29.749; *p* < 0.001, explaining an additional 14.2% variance in chronic fatigue (R^2^ change = 0.142; F(1,68) = 18.053; *p* < 0.001). Compared with no weekly visits, spending time in nature was associated with a reduction in chronic fatigue of 22.5 percentage points during the pandemic (see Table 5).

**Figure 5:**
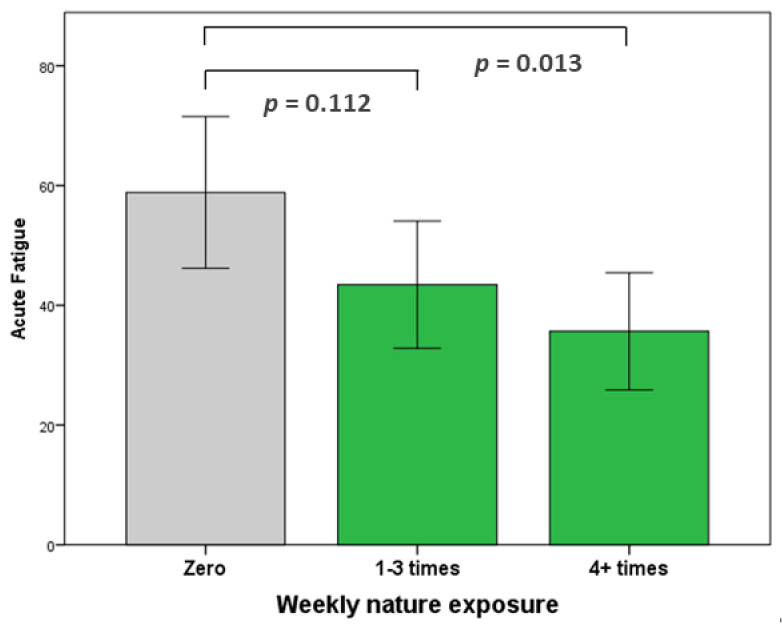
Acute fatigue during Covid-19. Drivers who visited nature 4+ times a week had significantly lower acute fatigue than those who did not visit nature at all. 95% confidence intervals.

A further ANOVA was used to investigate the effect of frequency of nature visits on acute fatigue. There was a statistically significant difference between the groups (F(2,68) = 4.418, *p* = 0.016). A Tukey post hoc test revealed that acute fatigue showed a non-significant trend for being lower in the 1–3 weekly visit group (43.45 ± 27.37, *p* = 0.112) and significantly lower in the 4+ weekly visits group (35.65 ± 22.62, *p* = 0.013) than for the no visits group (58.83 ± 27.06). No significant differences were observed between the 1–3 weekly visits and the 4+ weekly visits groups (see Figure 5).

An additional multiple regression was performed to consider the effect of the same potential confounding effects on acute fatigue. Only depression (*p* < 0.001) and time in nature (*p* < 0.001) contributed significantly to the prediction (note that due to the restrictions imposed on the study design by Covid-19, we were unable to repeat pre-Covid-19 measures of physical activity).

A hierarchical multiple regression was then conducted to investigate the ability of time in nature to predict acute fatigue, after controlling for depression. In the first step of the hierarchical multiple regression, depression was entered as a predictor. The model was statistically significant (F(1,69) = 25.910; *p* < 0.001), and explained 27.3% of the variance in acute fatigue (Table 6). After entry of time in nature at Step 2 the total variance explained by the model was 37.6% (F(2,68) = 20.487; *p* < 0.001). The introduction of time in nature explained an additional 10.3% of the variance in acute fatigue, after controlling for depression (R^2^ change = 0.102; F(1,68) = 11.225; *p* = 0.001). Compared with no weekly visits, spending time in nature was associated with a reduction in acute fatigue of 19.2 units during the pandemic (see Table 6).

## 4. Discussion

This study investigated the associations between exposure to nature and symptoms of acute and chronic fatigue, and anxiety and depression, in a sample of HGV drivers both prior to and during the Covid-19 pandemic. This work provides a novel contribution to our understanding of the potential therapeutic effects of nature exposure in a high-risk, and underserved, occupational group. After controlling for covariates, drivers who visited nature at least once a week exhibited 16% less chronic fatigue prior to the Covid-19 pandemic, and 23% less chronic fatigue and 20% less acute fatigue during the Covid-19 pandemic. No significant differences were observed for either symptoms of anxiety or depression and it is unclear, from this data, why this was the case.

Fatigue reduces cognitive function and increases the likelihood of crashes [27,28,71]. Fatigue is a predictor of physiological and psychological stress [22], is associated with decreased quality of life and increases morbidity and mortality across the general population [72]. From a physiological perspective, fatigue is linked with decreased cardiometabolic [18,32], cardiovascular [11,12,33] and musculoskeletal [15,16] health, as well as increased rates of cancer and all-cause mortality [34,35]. Fatigue is also closely linked to psychological disorders such as anxiety and depression, which can themselves negatively impact somatic health [45]. These health problems feature prominently among HGV drivers. Furthermore, drivers’ sedentary behaviour, combined with low levels of habitual physical activity and poor diet, results in disproportionately high rates of obesity. For example, 89% of the 386 drivers participating in the SHIFT study were overweight or obese at baseline, in comparison to 79% of men in England aged 45–54 years who were classified as overweight or obese in 2019 [73]. These factors combined increase the incidence of cardiovascular disease, cardiovascular mortality, musculoskeletal disorders, diabetes, cancer, cancer mortality and all-cause mortality in this occupational group [17,18,40,41,42,43,44].

The results presented here suggest that nature may provide a valuable, and low-cost and medication-free health and wellbeing intervention for HGV drivers. Despite the well-documented health consequences associated with this occupation, this at-risk cohort remain an underserved group in terms of health promotion efforts [74]. The current paper featured drivers enrolled in the SHIFT project, which aims to address this need by supporting drivers to take steps towards achieving a healthy lifestyle [1], although spending time in nature is currently not a feature of this particular intervention. Indeed, it was observed herein that participants in the control group reported a non-significant greater frequency of spending time in nature before and during the Covid-19 pandemic than drivers within the intervention group.

The reported association between time in nature and reduced chronic and acute fatigue has relevance beyond the logistics and transport industry. Fatigue is one of the most common complaints reported (by the general public) in primary care [75,76]. A recent study considering the general population (aged 45–86) in Lausanne (Switzerland) found that one in five individuals suffer from fatigue [77]. This figure is comparable to 22% [78], 22% [79], 38% [80] and 46.7% [81] reported in similar investigations (varying by patient age, geographic location and definition of fatigue). Common workplace practices, which span multiple industries, including those that involve shift work [82,83,84], are thought to contribute to worker fatigue. Shift work has become very common in modern society, with 76% of workers sampled from across the EU working ‘non-standard’ hours (i.e., outside of 730–1800, Monday to Friday) [85]. Workers in a range of areas, such as the medical [86], law enforcement [87] and construction [88] sectors, regularly work shifts, and would all benefit from interventions addressing the resultant fatigue.

A wealth of evidence has demonstrated the benefits of exposure to nature on human health and wellbeing [47,48,49]. These benefits have been recognised by the governments of Japan, Finland and South Korea, who currently fund public health initiatives promoting nature experiences as therapy [89,90]. However, there is currently insufficient empirical evidence to inform widespread adoption of this promising remedy, and nature prescriptions remain rare among medical professionals in the UK [91]. The primary explanation for this lack of scripting is that it remains unclear exactly which aspects of nature are responsible for the beneficial outcomes. Although it has been shown that, for example, visual stimulation [92,93], olfactory consumption of volatile organic compounds [94,95] and physical contact with wood [96] all promote positive outcomes, the precise mechanisms remain uncertain. The difficulty of identifying the relevant mechanisms lies in the fact that ‘Nature’ is tremendously complex and involves multi-species interaction.

Nevertheless, despite these challenges, significant steps have been taken toward understanding how to consume nature to derive benefit. White and colleagues recently identified the key dosage of nature exposure to be 120 min per week [97]. Participants (nearly 20,000 respondents to the UK’s Monitor of Engagement with the Natural Environment survey, representing both sexes, a range of ages and socioeconomic backgrounds) spending at least 120-min/week experienced consistently higher levels of health and wellbeing than those who did not spend time in nature on a weekly basis [97]. The present study adds to this earlier finding by revealing an association between one visit per week and decreased levels of both chronic and acute fatigue.

The main limitation of this study stems from its observational design. As a result, reverse causality cannot be ruled out. While the study findings are in-line with those reported in the literature on the health benefits of nature exposure, within the present sample an alternative explanation could be that drivers suffering from more fatigue were less likely to spend time in nature. Interventions are required to further examine the effect of nature exposure on levels of fatigue within this population. The hypothesis generated from this work is that nature exposure has beneficial effects in terms of reducing occupational fatigue in HGV drivers. An experimental trial would provide valuable evidence on the potential of this low-cost intervention to enhance driver health and wellbeing. A second limitation was imposed by the Covid-19 outbreak, which prevented the collection of device-based physical activity data during the pandemic. This limitation meant that data were not available that would allow for control of physical activity in the analyses, potentially exaggerating the effects of nature on fatigue during the pandemic. However, the pre-Covid-19 data did identify an effect of nature on fatigue, even after the effect of physical activity was removed. Finally, it has been suggested that self-assessment may be an insensitive indicator of fatigue [98]. While the measures of work-related chronic and acute fatigue used here were collected from a validated tool widely used to characterise occupational fatigue [63,64].

It is also acknowledged that these data were collected from a cohort of drivers participating in a health behaviour intervention. There is a chance therefore that the measures taken during the pandemic may be confounded by the fact that the drivers are enrolled in this trial, although the proportion of drivers within the intervention group spending time in nature before and during the pandemic remained constant at 67% during both time points. Furthermore, the associations observed between chronic fatigue and nature exposure from the baseline data support the findings seen during the pandemic, as the baseline data were collected prior to randomisation. It is notable that, as mentioned above, the SHIFT intervention does not currently include any content regarding nature exposure; it is an educational based intervention focusing on physical activity promotion and the adoption of a healthy diet.

Despite these limitations, this study provides novel evidence of the associations between nature exposure and acute and chronic fatigue in a sample of HGV drivers. Strengths of this study include its focus on this traditionally hard to reach, and at risk, occupational group who saw an increase in the demands of their job during the pandemic. The comprehensive and robust measures taken at baseline of the main RCT enabled the observations seen during the pandemic to be tested during an earlier time point, whilst controlling for a number of covariates. Whilst the completion rate of the questionnaire during the pandemic (40%) was modest, this subsample of the wider ‘SHIFT’ cohort included in these analyses did not differ significantly from the remainder of the sample at baseline. This suggests that the participants included herein are largely representative of the sample participating in the wider study, which in turn is also largely representative of the characteristics of HGV drivers in the UK, in terms of sex distribution and average age [99].

## 5. Conclusions

This work revealed a significant difference in both acute and chronic fatigue between drivers who visited nature at least once a week and those who did not. As fatigue has a range of physical and mental health sequelae, nature may make a valuable contribution to interventions to promote the health and wellbeing of this underserved group. To our knowledge, this is the first study linking time in nature to occupational health in drivers. Further, experimental, work is required to improve our understanding of the mechanism underpinning the widely observed relationship between nature and human health and wellbeing.

## Figures and Tables

**Table 1:** Descriptive statistics of the study sample at baseline (98.9% male). BMI, body mass index; HGV, heavy goods vehicle.

Participant Characteristics	*n*	Mean (SD)
Age (y)	89	49.9 (8.9)
Height (cm)	88	178.6 (6.7)
Weight (kg)	88	94.9 (14.6)
BMI (kgm^−2^)	88	29.9 (4.7)
Working hours per week	89	46.6 (8.4)
Years as HGV driver	89	18.3 (10.6)

**Table 2:** Comparison of key outcome variables between participants who made weekly visits to nature, and those who did not. The results for chronic fatigue remain significant after applying a Bonferroni correction changes α to 0.05 ÷ 4 = 0.0125. HADS, Hospital Anxiety and Depression Scale.

	Do You Spend Time in Nature at Least Once a Week?		Comparison of Nature vs. No Nature	
	Yes (*n* = 64) Mean (SD)	No (*n* = 25) Mean (SD)	*t*	*p*
Chronic fatigue	27.40 (23.63)	42.53 (24.86)	−2.888	0.005
Acute fatigue	44.23 (21.86)	50.53 (21.03)	−1.197	0.235
HADS Anxiety	4.45 (2.85)	5.04 (2.98)	−0.863	0.390
HADS Depression	7.84 (1.61)	8.16 (1.38)	0.868	0.388

**Table 3:** Hierarchical multiple regression model of chronic fatigue. Time in nature is a significant predictor of chronic fatigue after controlling for anxiety.

	R	R^2^	R^2^ Change	B	SE	Beta	t
**Step 1**	0.542	0.294 ***					
Weekly hours				0.648	0.261	0.459 *	2.480
Anxiety				3.697	0.778	0.469 ***	4.754
Step count				0.004	0.003	0.591	1.480
MVPA				−0.522	0.298	−0.289	−1.753
LPA				−18.294	14.826	−0.549	−1.234
**Step 2**	0.617	0.381 **	0.087				
Weekly hours				0.679	0.247	0.251 **	2.752
Anxiety				3.457	0.737	0.429 ***	4.693
Step count				0.006	0.003	0.864 *	−2.342
MVPA				−0.665	0.284	−0.368 *	−2.342
LPA				−30.405	14.447	−0.912 *	−2.105
Time in nature				−15.822	4.787	−0.311 **	−3.305

Note. Statistical significance: * *p* < 0.05; ** *p* < 0.01; *** *p* < 0.001.

**Table 4:** Comparison of key outcome variables between participants who made weekly visits to nature, and those who did not. The results for chronic and acute fatigue remain significant after applying a Bonferroni correction changes α to 0.05 ÷ 4 = 0.0125.

	Do You Spend Time in Nature at Least Once a Week?		Comparison of Nature vs. No Nature	
	Yes (*n* = 51) Mean (SD)	No (*n* = 21) Mean (SD)	*t*	*p*
Chronic fatigue	25.95 (23.63)	49.83 (28.29)	−3.621	0.001
Acute fatigue	39.94 (25.40)	58.83 (27.06)	−2.769	0.007
HADS Anxiety	4.08 (4.00)	4.45 (4.30)	−0.345	0.731
HADS Depression	3.43 (4.33)	3.35 (3.62)	0.074	0.941

**Table 5:** Hierarchical multiple regression model of chronic fatigue. Time in nature is a significant predictor of chronic fatigue after controlling for anxiety.

	R	R^2^	R^2^ Change	B	SE	Beta	t
**Step 1**	0.570	0.325 ***					
Anxiety				3.805	0.660	0.570 ***	5.765
**Step 2**	0.683	0.467 **	0.142				
Anxiety				3.700	0.591	0.555 ***	6.256
Time in nature				−22.511	5.298	−0.377 **	−4.249

Note. Statistical significance: ** *p* < 0.01; *** *p* < 0.001.

**Table 6:** Hierarchical multiple regression model of acute fatigue. Time in nature is a significant predictor of acute fatigue after controlling for depression.

	R	R^2^	R^2^ Change	B	SE	Beta	t
**Step 1**	0.522	0.273 ***					
Depression				3.438	0.675	0.522 ***	5.090
**Step 2**	0.613	0.376 **	0.103				
Anxiety				3.457	0.630	0.525 ***	5.484
Time in nature				−19.180	5.725	−0.350 **	−3.350

Note. Statistical significance: ** *p* < 0.01; *** *p* < 0.001.

## Data Availability

Data can be made available upon request.
